# Exposed Nerves

**Published:** 1874-07

**Authors:** C. W. Westmoreland

**Affiliations:** Columbus, Miss.


### EXPOSED NERVES.

BY C. W. WESTMORELAND, D. D. S., COLUMBUS, MISS.

I had prepared something on another subject for the consideration of the readers of the Register, but my ill success in the extraction of three teeth recently has induced me (more for my own information and the comfort of my patients than any thing else) to submit this article on a hackneyed subject. And in passing, allow me to say, that I fear almost all articles appearing in our journals report successes and never failures. I am going to report some failures. Did you ever noticed this peculiarity about teeth, that some days when you are expecting that this weather will surely produce tooth-ache, that not a case of it is found, and at some other time, four or five or it may be a dozen will come when you least expect it.

This is my experience. Occasionally I get in the way of breaking them off. Two weeks ago I tried to extract teeth for three different patients, and crushed all three teeth, leaving the nerves protruding from all of them. I am usually successful and could have succeeded in these cases if the patients would have submitted. They would not and the question was, how to destroy these nerves. Of course the usual nerve paste could not be retained at its place for a long enough time to destroy them. Well, the something needed ought to act instantaneously. I had tried nitric acid before, but was not successful. I had also used nitrate of mercury, a powerfnl escarotic, with equally poor success. Well chromic acid (easier to apply than either of the above on account of its crystalline structure) did not destroy them. Concentrated carbolic acid ought to devitalize them, but I found nothing that would produce the desired effect. And in my opinion nothing short of actual cautery with a hot wire will destroy a nerve in this condition. I venture to say that not a reader of this article, who has extracted many teeth, but has found himself in this dilemma. For the present, I will await the information which you or any interested may be willing to give.

